# *Chlorella zofingiensis* as an Alternative Microalgal Producer of Astaxanthin: Biology and Industrial Potential

**DOI:** 10.3390/md12063487

**Published:** 2014-06-10

**Authors:** Jin Liu, Zheng Sun, Henri Gerken, Zheng Liu, Yue Jiang, Feng Chen

**Affiliations:** 1 Institute for Food & Bioresource Engineering, College of Engineering, Peking University, Beijing 100871, China; 2 Institute of Marine and Environmental Technology, University of Maryland Center for Environmental Science, Baltimore, MD 21202, USA; 3 College of Fisheries and Life Science, Shanghai Ocean University, Shanghai 201306, China; E-Mail: zsun@shou.edu.cn; 4 Department of Applied Sciences and Mathematics, Arizona State University Polytechnic campus, Mesa, AZ 85212, USA; E-Mail: hgerken@asu.edu; 5 Department of Biochemistry and Molecular Biophysics, Columbia University, New York, NY 10032, USA; E-Mail: zl2354@columbia.edu; 6 School of Food Science, Jiangnan University, Wuxi 214122, China; E-Mail: yjiang@hotmail.com.hk

**Keywords:** astaxanthin, *Chlorella zofingiensis*, fed-batch, genetic engineering, mass cultivation, microalgae, stress

## Abstract

Astaxanthin (3,3′-dihydroxy-β,β-carotene-4,4′-dione), a high-value ketocarotenoid with a broad range of applications in food, feed, nutraceutical, and pharmaceutical industries, has been gaining great attention from science and the public in recent years. The green microalgae *Haematococcus pluvialis* and *Chlorella zofingiensis* represent the most promising producers of natural astaxanthin. Although *H. pluvialis* possesses the highest intracellular astaxanthin content and is now believed to be a good producer of astaxanthin, it has intrinsic shortcomings such as slow growth rate, low biomass yield, and a high light requirement. In contrast, *C. zofingiensis* grows fast phototrophically, heterotrophically and mixtrophically, is easy to be cultured and scaled up both indoors and outdoors, and can achieve ultrahigh cell densities. These robust biotechnological traits provide *C. zofingiensis* with high potential to be a better organism than *H. pluvialis* for mass astaxanthin production. This review aims to provide an overview of the biology and industrial potential of *C. zofingiensis* as an alternative astaxanthin producer. The path forward for further expansion of the astaxanthin production from *C. zofingiensis* with respect to both challenges and opportunities is also discussed.

## 1. Introduction

**Figure 1:**
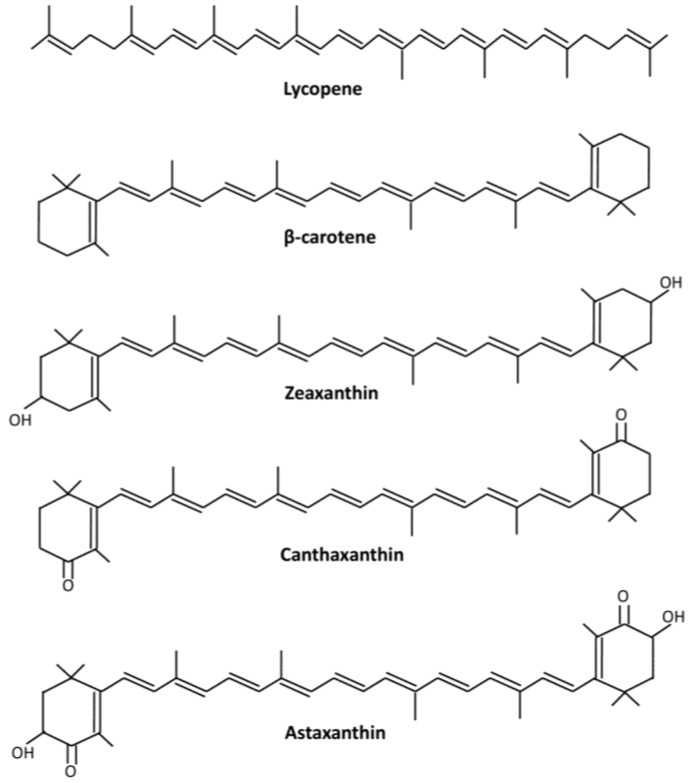
Chemical structure of selected carotenoids.

Microalgae are sunlight-driven cell factories that are able to efficiently utilize carbon dioxide for the production of numerous products such as proteins, polysaccharides, oils, vitamins, carotenoids and other biologically active compounds, and are therefore used in a wide variety of technological applications for the development of feed and food products [1,2]. Microalgae-based carotenoid production has been intensively studied in recent years, e.g., β-carotene from *Dunaliella salina* [3], zeaxanthin from *Synechocystis* [4], lutein from *Chlorella protothecoides* [5], and astaxanthin from *Haematococcus pluvialis* and *Chlorella zofingiensis* [6,7]. Carotenoids represent a group of structurally diverse terpenoid pigments with the majority being a 40-carbon chain conjugated by double bonds (Figure 1). Of the carotenoids, astaxanthin, a red ketocarotenoid, has the highest value. Because of its strong pigmentation function, powerful antioxidative activity and broad beneficial effects on human health, astaxanthin possesses a wide range of applications in feed, food, nutraceutical, and pharmaceutical industries [8,9,10]. *C. zofingiensis* grows fast phototrophically, heterotrophically and mixtrophically, is easy to be cultured and scaled up both indoors and outdoors, achieves ultrahigh cell density, and is therefore regarded as a potential alternative to *H. pluvialis* to produce astaxanthin [7,11,12,13,14]. This article intends to provide an overview of the current status of astaxanthin and the potential of using *C. zofingiensis* for astaxanthin production. The possible improvements in production economics are also discussed, including genetic engineering for strain optimization, exploration of advanced culture systems and biorefinery-based production strategies. Breakthroughs and innovations occurring in these areas will greatly expand the production capacity and lower the production cost, driving *C. zofingiensis* into the high-value market for cost-effective production of astaxanthin.

## 2. Astaxanthin and Its Applications

Astaxanthin has a chemical formula of C_40_H_52_O_4_ and a molecular weight of 596.86 in geometric *cis* and *trans* isomers; the latter is thermodynamically more stable than the former and is found predominantly in nature [15]. *Trans* astaxanthin has three forms of stereoisomers: two enantiomers (3*R*, 3′*R* and 3*S*, 3′*S*) and a meso form (3*R*, 3′*S*) [16]. The isomers are identical in molecular and structural formula with the only difference in position of the rotating functional group. 3*S*, 3′*S* is the most abundant isomer in nature, predominantly synthesized by green microalgae [17]. Synthetic astaxanthin contains a mixture of isomers (3*S*, 3′*S*), (3*S*, 3′*R*), and (3*R*, 3′*R*) at the ratio of 1:2:1. Astaxanthin is either in free form or esterified on its one or both hydroxyl groups with various fatty acids such as palmitic, stearic, and oleic acids. Synthetic astaxanthin is in free form while the natural one found in algae is mainly in esterified form.

A strong pigmentation allows astaxanthin to serve as a feed supplement for aquaculture. The use of astaxanthin in aquaculture species, for example salmon and lobster, has been extensively studied [18,19,20]. Animals cannot synthesize carotenoids *de novo* but have to obtain carotenoids through their food chain or feeds. The dietary carotenoids give such organisms as salmonids and crustaceans the reddish-orange color that is regarded by consumers as one of the key quality attributes. Astaxanthin is the major carotenoid found in certain marine animals, for example, in crab, the red pigment accounts for more than 80% of total carotenoids [21]. Because of the higher color intensity and better absorption by the digestive tract of salmonids, astaxanthin is preferred over canthaxanthin in aquatic farming [22]. In addition to pigmentation, astaxanthin has been demonstrated to benefit the growth and survival of larval fish and shrimp [23,24]. Astaxanthin is also used in the tropical marine industry aiming to pigment ornamental fish [25].

Furthermore, astaxanthin possesses potent antioxidant activity, which has been demonstrated in numerous studies [26,27,28,29,30,31,32]. Astaxanthin is much more effective in scavenging free radicals than other carotenoids and Vitamin E [26,27,29]. It is the powerful antioxidative activity that makes astaxanthin beneficial to human health, e.g., reducing DNA damage [33], protecting cells of eyes and skin from UV-light mediated photo-oxidation [34], attenuating inflammation by quenching ROS [35], boosting immune system by enhancing the production of antibodies and increasing the total number of T-cells [36,37], and benefiting heart health by modifying blood levels of LDL and HDL cholesterol [38].

## 3. Sources of Astaxanthin

Being widely used in aquatic farming as a feed additive, the astaxanthin market in the United States is estimated to be US$200 million per year at a price of around US$2,500 per kg. Nowadays, commercial astaxanthin for aquaculture is mainly chemically synthesized from petrochemical sources, whereas natural astaxanthin contributes only to a minor portion of the market [8]. Although the current astaxanthin market is dominated by synthetic products, the growing demand of customers for natural foods has urged the production of astaxanthin from natural sources such as crustacean by-products, yeast, microalgae and transgenic plants.

Crustacean by-products such as heads, shells and tails generated from food processing consist mainly of chitin, proteins, fatty acids and carotenoids [39]. The carotenoid composition and content in crustacean by-products have been well studied [21,40,41,42,43,44], and indicate that there is potential for these by-products to be a natural astaxanthin source. However, these by-products generally contain only a small amount of astaxanthin but high contents of ash and chitin that significantly decrease the digestibility [16], making the use of crustacean by-products in fish feeding less feasible and economical.

The red yeast *Xanthophyllomyces dendrorhous* (previously referred as *Phaffia rhodozyma*) is able to accumulate astaxanthin [45,46,47,48]. *X. dendrorhous* can grow fast and achieve high cell density by utilizing a variety of carbon sources such as glucose, xylose and even molasses. However, the cellular astaxanthin content is relatively low, varying from 0.16 to 1.1 mg·g^−1^ dry weight depending on different strains of *X. dendrorhous* [49]. The astaxanthin content can be enhanced by using genetic modification strategies [50], which allowed the isolated mutants of *X. dendrorhous* to produce 1.5–3.8-fold more astaxanthin than the wild type cells. Currently, *X. dendrorhous* is sold commercially in a fine powder as a natural source of astaxanthin as fish feed. The thick cell walls of yeast, however, hinder the assimilation of astaxanthin by fish and thus cell wall disruption is needed [51]. The *X. dendrorhous* derived astaxanthin is in the 3*R*, 3′*R* form which is exceptional among known astaxanthin producing organisms [52].

With the exception of *Adonis annua*, which produces astaxanthin in its flower petals [53], higher plants are unable to synthesize astaxanthin because they are devoid of the β-carotene ketolase that induces keto-moieties to the 4,4′ position of β-ionone rings of β-carotene and zeaxanthin. However, the production of astaxanthin can be achieved through the functional expression of a heterologous β-carotene ketolase gene in plants. There are numerous studies addressing the accumulation of astaxanthin in transgenic plants including tobacco [54,55,56,57], *Arabidopsis* [58], potato [59,60], carrot [61] and even tomato [62]. Although astaxanthin is produced in low amounts in transgenic plants, the accumulated astaxanthin adds nutritional value to the edible parts of crop plants for human health and may reach commercial use in the future.

Microalgae represent a source with the most potential for natural astaxanthin production and have therefore been subjected to thorough investigations in terms of strain screening, culture medium optimization, stress induction, cultivation strategy, modification and genetic improvements for astaxanthin production [6,7,12,13,63,64,65,66,67,68,69]. The ability of *H. pluvialis* to accumulate astaxanthin up to 4% of its dry biomass, the highest known content in nature, has led researchers to focus on *H. pluvialis* for commercial astaxanthin production [63]. *Haematococcus* algal meal has been approved as a color additive in salmonid feeds and as a dietary-supplement for human consumption in Japan, USA and some other countries. In large-scale, enclosed photobioreactor or outdoor systems, a two-step process is employed for the production of astaxanthin-rich *Heamatococcus* cells: cell biomass accumulation and astaxanthin induction. First, vegetative cells accumulate and achieve a sufficient density under optimal growth conditions; the high density culture is then subjected to stress conditions (e.g., deprivation of nitrate, high light intensity, and salt stress) for astaxanthin induction [6,70].

Although *H. pluvialis* can accumulate a high amount of astaxanthin, it grows relatively slowly with a low biomass yield and is susceptible to contamination by other fast-growing organisms [17,64,67,71]. In addition, extremely high light illumination is required for astaxanthin induction and accumulation in this alga thus hindering its commercial application [70,72]. The green alga *Chlorella zofingiensis* has recently been regarded as a potential alternative host for mass production of astaxanthin due to its fast growth, low sensitivity to contamination and unfavorable environments, and astaxanthin accumulation under heterotrophic conditions with glucose as the sole carbon and energy source [7,11,12,13,66].

Despite the many natural sources of astaxanthin, the majority of astaxanthin on the market is synthetically derived. The difference between natural and synthetic astaxanthin lies in their stereochemical orientation. Synthetic astaxanthin is a mixture of three isomeric forms with 50% being 3*R*, 3′*S*, while natural astaxanthin from microalgae is in the 3*S*, 3′*S* form. The 3*S*, 3′*S* form of astaxanthin is reported to give a stronger pigmentation to rainbow trout than other forms and is therefore preferred as a feed additive for fish farming [73]. *Haematococcus* astaxanthin is thought to play important roles in human health and nutrition, while the biological effect of other isomers has not been established [8]. Moreover, unlike synthetic astaxanthin that is present in free form, natural astaxanthin usually exists mono-esterified or di-esterified with fatty acids [7,74,75]. The esterified astaxanthin is inherently more stable than the free form, thus providing a greater shelf life without oxidation. As consumers become more and more aware of the putative benefits of natural astaxanthin, and as the commercial production is optimized with lowered costs, the natural astaxanthin will beat petroleum derived synthetic astaxanthin and finally dominate the market.

## 4. *Chlorella zofingiensis* as a Potential Producer of Astaxanthin

### 4.1. Taxonomy

**Figure 2:**
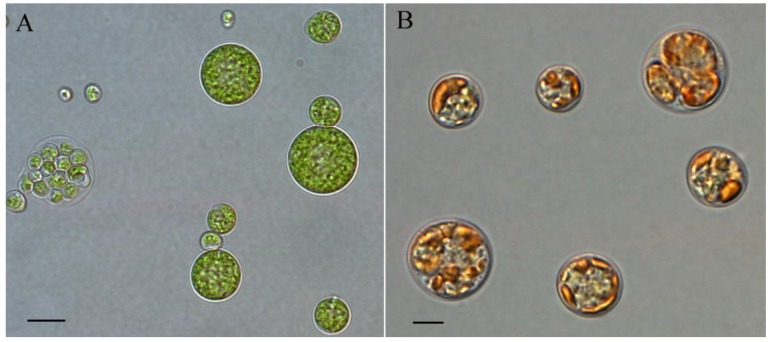
Light micrographs of *C. zofingiensis* cells under favorable (**A**) and stress (**B**) conditions. Bars, 5 μm.

*C. zofingiensis* is a fresh water green microalga once assigned to the Genus *Chlorella*, but some reports classify this alga into Genus *Muriella* [76], *Mychonastes* [77], or *Chromochloris* [78], indicating an evolutionary distance from the “true *Chlorella*”. *C. zofingiensis* cells are non-motile and in unicellular and spherical form, with the cell size ranging from 2 to 15 μm in diameter (Figure 2). Through the formation of autospores, *C. zofingiensis* asexually reproduces daughter cells from non-motile parental cells [79]. The asexual reproduction generally involves three phases: growth, ripening, and division [80].

**Figure 3:**
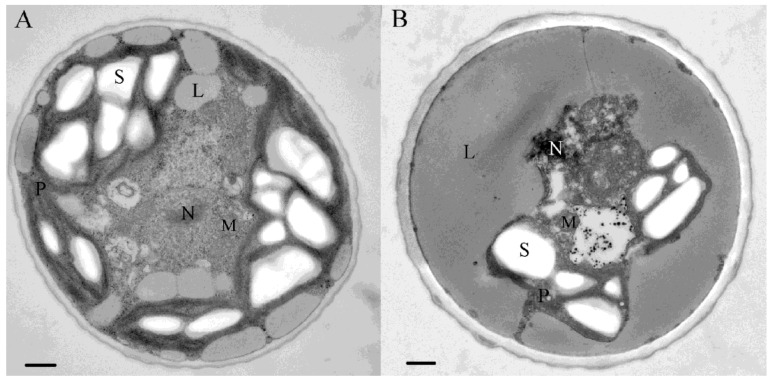
The ultrastructure of *C. zofingiensis* cells under favorable (**A**) and stress (**B**) conditions. Bars, 0.5 μm;·L, lipid body; N, nucleus; M, mitochondria; P, plastid; S, starch granule.

Observations using an electron microscope reveal that under favorable culture conditions, *C. zofingiensis* has a cup-shaped chloroplast in which starch is abundant in scattered granules (Figure 3A). The chloroplast situates peripherally in the cytoplasm and occupies about half of the cell volume; in contrast, the nucleus is somewhat centrally located in the cytoplasm (Figure 3A). Mitochondria appear in an ovoid shape and are closely associated with chloroplast (Figure 3A). Lipid bodies are also observed peripherally and can be induced to build up under stress conditions, which may merge together to form a thick layer surrounding the shrunken chloroplast which has decreased starch levels (Figure 3B).

### 4.2. Pigment Profile

**Figure 4:**
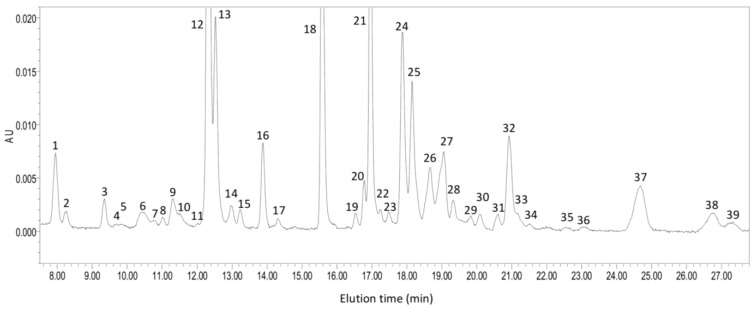
A HPLC chromatogram showing a general pigment profile of *C. zofingiensis* under astaxanthin-inducing conditions (four-day induction with 20 g L^−1^ glucose in the dark). 1 Neoxanthin; 2 Violaxanthin; 3 Antheraxanthin; 4, 5, 7, 8, 11, 19, 28, 29, 30, 35, 36 Unknown or degraded lutein and chlorophylls; 6 Astaxanthin; 9, 10 Adonixanthin; 12 Lutein; 13, 14, 15 Zeaxanthin; 16, 17 Canthaxanthin; 18 Chlorophyll b; 20, 22 β-Cryptoxanthin; 21 Chlorophyll a; 23 Echinenone; 24, 26 Astaxanthin mono-ester; 25, 27 Adonixanthin mono-ester; 31 α-Carotene; 32, 33, 34 β-Carotene; 37, 39 Astaxanthin di-ester; 38 Adonixanthin di-ester.

*C. zofingiensis* contains chlorophylls a and b for photosynthesis and both primary and secondary carotenoids are observed within *C. zofingiensis* cells. Like plants and other algae, *C. zofingiensis* synthesizes and accumulates primary carotenoids (e.g., β-carotene, lutein and zeaxanthin) in the chloroplast [81,82]. In contrast, secondary carotenoids such as astaxanthin, canthaxanthin and adonixanthin are found to accumulate in lipid bodies outside the chloroplast [11,81]. The accumulation of secondary carotenoids, in general, is considered to be associated with stress conditions, under which algal cells are protected against oxidative damage by these anti-oxidative carotenoids through quenching the excessive reactive oxygen species (ROS) and other free radicals [81,83]. For example, when growing under high light and nitrogen starvation, *C. zofingiensis* produces mainly secondary carotenoids, of which around 70% is astaxanthin [81]. A general pigment profile of *C. zofingiensis* under astaxanthin-inducing conditions is shown in Figure 4. Astaxanthin in *C. zofingiensis* occurs predominantly in the form of mono- and di-esters.

### 4.3. Carotenoid Biosynthesis

**Figure 5:**
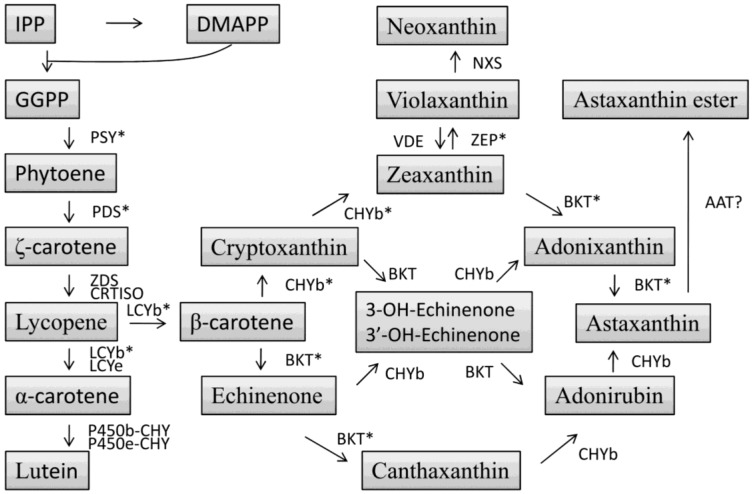
Schematic diagram of possible carotenoid biosynthesis in microalgae. IPP, isopentenyl pyrophosphate; DMAPP, dimethylallyl pyrophosphate; GGPP, geranylgeranyl pyrophosphate; PSY, phytoene synthase; PDS, phytoenedesaturase; ZDS, ζ-carotene desaturase; CRTISO, carotene isomerase; LCYb, lycopene β-cyclase; LCYe, lycopene ε-cyclase; P450b-CHY, cytochrome P450 β-hydroxylase; P450e-CHY, cytochrome P450 ε-hydroxylase; CHYb, β-carotene hydroxylase; BKT, β-caroteneketolase; ZEP, zeaxanthinepoxidase; VDE, violaxanthin de-epoxidase; NXS, neoxanthin synthase; AAT, astaxanthin acyl transferase; * denote the genes characterized in *C. zofigiensis*.

The biochemical and molecular aspects of carotenoid biosynthesis in higher plants have been well elucidated [84], but remain relatively poorly understood in microalgae. However, it is widely accepted that microalgae follow a pathway similar to higher plants for the biosynthesis of carotenoids, which generally involves five types of reactions (Figure 5): (1) formation of isopentenyl diphosphate (IPP); (2) condensation of isoprene units resulting in phytoene, the first C_40_ carotene; (3) four step-wise desaturation reactions converting colorless phytoene to pink-colored lycopene; (4) cyclization of lycopene to form β-carotene and α-carotene; and (5) synthesis of xanthophylls by the introduction of oxygen groups to carotenes.

In eukaryotes, IPP is the first intermediate in the carotenoid biosynthetic pathway, which may be either assembled from three molecules of acetyl-CoA via mevalonate (mevalonate pathway) or condensed from pyruvate and glyceraldehyde phosphate (mevalonate independent pathway). *C. zofingiensis* is thought to synthesize IPP via mevalonate independent pathway as *H. pluvialis* does; however, this remains to be experimentally demonstrated. Sequential addition of three molecules of IPP to dimethylallyl-diphosphate (DMAPP), an isoform of IPP, results in the formation of the C_20_ compound geranylgeranyl pyrophosphate (GGPP), which is catalyzed by GGPP synthase (GGPPS), one member of a closely related family of prenyl transferases (Figure 5). Two GGPP molecules are then condensed head-to-head to form phytoene, the first committed step of carotenoid biosynthetic pathway, catalyzed by phytoene synthase (PSY) that is regarded as a key rate-limiting enzyme controlling the carbon flux into the pathway [85,86]. The *PSY* gene from *C. zofingiensis* contains an open reading frame (ORF) of 1263 bp that encodes a putative protein of 420 amino acids [85]. *C. zofingiensis PSY* harbors three introns and is present as a single copy in the genome [85]. Heterologous overexpression of the *C. zofingiensis PSY* gene in *Chlamydomonas reinhardtii* can enhance the intracellular accumulation of carotenoids [85], which is consistent with previous studies overexpressing *PSY* in algae or higher plants [85,86,87,88].

**Figure 6:**
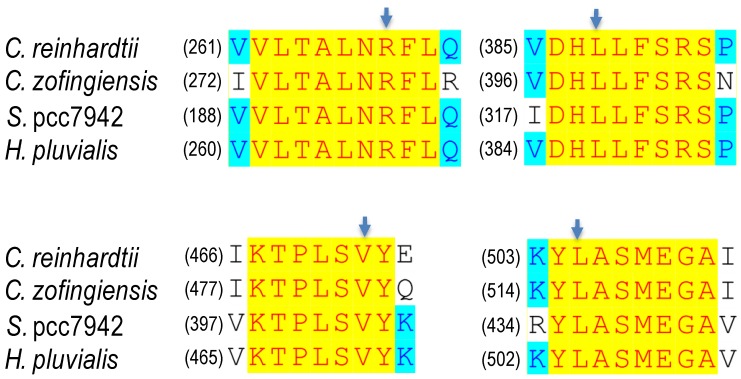
Point mutations that affect desaturation activity and norflurazon resistance of PDS. *C. reinhardtii*, *Chlamydomonas reinhardtii*; *C. zofingiensis*, *Chlorella zofingiensis*; *S*. pcc7942, *Synechococcus* pcc7942, *H. pluvialis*, *Haematococcus pluvialis*. Arrows indicate the positions of point mutations.

The synthesis of lycopene from phytoene requires four step-wise desaturations, involving two structurally and functionally related enzymes phytoene desaturase (PDS) and ζ-carotene desaturase (ZDS) (Figure 5). PDS is responsible for the first two desaturation steps resulting in the formation of ζ-carotene, which is further desaturated by ZDS to lycopene. *PDS* from *C. zofingiensis* has been cloned and characterized [89]. The ORF of this gene, interrupted by six introns, encodes a polypeptide of 558 amino acid residues; similar to *PSY*, *C. zofingiensis* genome contains only a single copy of *PDS* [89]. There are several conserved motifs thought to be important to the desaturation activity of PDS (Figure 6). Point mutations in these motifs have been demonstrated to alter, to various extents, the desaturation activity of PDSs from *Synechococcus* pcc7942, *H. pluvialis*, *C. zofingiensis*, and *C. reinhardtii* (Table 1). In *C. zofingiensis* PDS, for example, the mutations of R279P, L399P, V483G, and L516R attenuate the desaturation activity by up to 69%. Intriguingly, an L–F change at position 516 enhances the desaturation activity by 30%, compared to the 23% decrease caused by the L–R exchange. Surrounding the L residue (503–528) is a conserved motif present in PDS polypeptides of higher plants, algae and cyanobacteria, which is a proposed carotenoid binding site [90]. The L–F exchange may benefit the kinetics of phytoene binding to this enzyme, leading to the enhanced desaturation activity. Co-factors are required to facilitate the desaturation activity [91,92]. Therefore, it is also possible that the exchange enhances the specific binding of co-factors, contributing to the increased PDS activity. PDS is also considered as a rate-limiting enzyme in carotenoid biosynthesis [93]. Overexpression of a bacterial phytoene desaturase gene in tobacco [94] and tomato [95], and an endogenous *PDS* gene in *H. pluvialis* [96], *C. reinhardtii* [92], and *C. zofingiensis* [69] have been reported to promote carotenoid synthesis.

**Table 1:** The effect of point mutations on PDS desaturation activity and norflurazon resistance.

Organism	Mutation	Desaturation Activity (%) ^a^	Norflurazon Resistance ^b^	Reference
*C. zofingiensis*	R279P L399P V483G L516R L516F	32 94 81 67 133	18 14 17 28 31	[69]
*H. pluvialis*	L504R	<100	43	[96]
*C. reinhardtii*	L505R L505F	80 128	24 29	[92]
*S*. pcc7942	R195P L320P V403G L436R	24 91 83 67	23 8 15 76	[93]

^a^ relative *in vitro* desaturation activity compared to wild type PDS, which is set as 100%; ^b^ relative norflurazon resistance compared to wild type PDS, which is set as 1.

**Figure 7:**
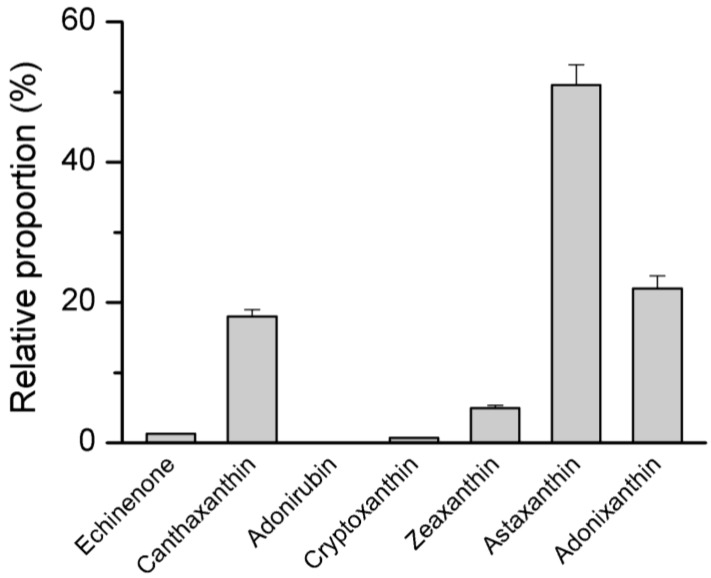
The relative proportion of carotenoids in *C. zofingiensis* under astaxanthin induction conditions (eight-day induction with 20 g·L^−1^ glucose in the dark). Each carotenoid is expressed as percentage of the sum of echinenone, canthaxanthin, adonirubin, cryptoxanthin, zeaxanthin, adonixanthin, and astaxanthin, which is set as 100%. Adonirubin is not detected.

Once synthesized, lycopene has two potential destinations; it can be cyclized to α-carotene or β-carotene. α-carotene has a β-ring and a ε-ring on each side which differentiates it from β-carotene, which harbors two β-rings end groups on both sides. Lycopene β-cyclase is responsible for the formation of β-carotene, which has been characterized from *C. zofingiensis* [97]. β-carotene serves as the direct precursor for astaxanthin biosynthesis, via a set of hydroxylation and ketolation steps and there are multiple routes it can follow to reach astaxanthin (Figure 5). In *H. pluvialis*, it is thought that β-carotene is converted to canthaxanthin via a two-step ketolation catalyzed by β-carotene ketolase (BKT), followed by a further two-step hydroxylation by β-carotene hydroxylase (CHYb) for the formation of astaxanthin [63]. In contrast to *H. pluvialis*, *C. zofingiensis* seems to employ an alternative pathway for astaxanthin biosynthesis. Under astaxanthin induction conditions, *C. zofingiensis* accumulates mainly astaxanthin, followed by adonixanthin, canthaxanthin, and zeaxanthin, and trace amount of echinenone and cryptoxanthin (Figure 7). Based on these data, one may infer that astaxanthin is synthesized from the ketolation of zeaxanthin instead of the hydroxylation of canthaxanthin, which appears unable to be converted to astaxanthin causing it to accumulate as an end product. This hypothesis is partially supported by a study using the BKT inhibitor diphenylamine (DPA) [98]. When exposing *C. zofingiensis* to astaxanthin induction conditions, the addition of DPA resulted in an increase in the intracellular accumulation of zeaxanthin at the cost of adonixanthin and astaxanthin. The further confirmation of this hypothesis, however, requires the support of *in vitro* assay data. *C. zofingiensis BKT* and *CHYb* genes have been cloned and characterized [83,99]. Each of the two genes is present as a single copy in *C. zofingiensis* genome. We have recently performed the *in vitro* enzymatic assays of BKT and CHYb and the data indicated that BKT was able to catalyze the ketolation of β-carotene to canthaxanthin and of zeaxanthin to astaxanthin, and CHYb showed the ability to hydroxylate β-carotene to zeaxanthin but not canthaxanthin to astaxanthin [100], further supporting that astaxanthin synthesis in *C. zofingiensis* is derived from zeaxanthin rather than canthaxanthin. Although BKT is capable of converting zeaxanthin to astaxanthin, it has a relatively low ketolation activity, causing the accumulation of the intermediate adonixanthin (Figure 7). Astaxanthin is present predominantly in the form of esters in *C. zofingiensis* [32]. The esterification of astaxanthin with fatty acids may need an acyltransferase; however, this acyltransferase has not been identified either in *C. zofingiensis* or in *H. pluvialis*.

Although astaxanthin is believed to accumulate in lipid bodies (LBs), the location for synthesis remains unclear. It is hypothesized in *H. pluvialis* that the chloroplast-localized β-carotene serves as an intermediate which can cross the plastid membranes towards cytoplasmic LBs where BKT and CHYb enzymes are available to convert β-carotene to astaxanthin [82,101]. This membrane-crossing process may be mediated by carotenoid-binding proteins instead of the vesicular transport as the latter is not observed with ultrastructural studies [101]. The inhibition of *de novo* fatty acid synthesis is accompanied by a drastic decrease in astaxanthin accumulation in *H. pluvialis* [102]. In contrast, *C. zofingiensis* produces more astaxanthin upon inhibition of the *de novo* fatty acid synthesis [100]. We infer that in *C. zofingiensis*, unlike in *H. pluviali*s, the esterification of astaxanthin may not require the *de novo* synthesized fatty acids, but rather, utilizes the fatty acids released from the turnover of chloroplast membrane lipids.

### 4.4. Factors Affecting Growth and Astaxanthin Production

Generally, microalgal reproduction requires light, carbon, nitrogen, phosphorus, inorganic salts, water, *etc.* The growth, astaxanthin content and astaxanthin productivity of *C. zofingiensis* are culture condition-dependent and can be greatly influenced by a variety of nutrient and environmental factors.

#### 4.4.1. Nutrients

Carbon is the most prominent element of microalgae that accounts for approximately 50% of the microalgal biomass. Carbon dioxide is the primary inorganic carbon source for photosynthesis of *C. zofingiensis*. Under photoautotrophic growth conditions, CO_2_ concentration has a crucial impact on the growth of *C. zofingiensis*. Too low or too high CO_2_ is suboptimal for the growth of *C. zofingiensis*. Low CO_2_ is unable to provide enough carbon to support efficient photosynthesis while high CO_2_ causes a pH decrease in the culture medium, which may in turn inhibit the algal growth. Accordingly, air enriched with 1%–5% CO_2_ is provided for the efficient growth of *C. zofingiensis*. In addition, *C. zofingiensis* is capable of using organic carbon sources such as sugar and acetate. Liu *et al.* [103] investigated the effect of various monosaccharides and disaccharides on growth of *C. zofingiensis*, and the results indicated that glucose, fructose, mannose and sucrose were efficiently consumed by the alga for rapid growth whereas lactose and galactose were poorly assimilated and could not support robust algal growth. Accordingly, glucose gave rise to the highest astaxanthin content while lactose led to the lowest astaxanthin content [13]. Under heterotrophic conditions with glucose as the sole carbon source, the cell density and astaxanthin content of *C. zofingiensis* is associated with the initial glucose concentration. Increasing the initial glucose concentration leads to greater final cell densities until the glucose is over 30 g·L^−1^ when the increase of glucose concentration causes no significant increase in cell density, but rather, decreases the specific growth rate [7,12]. This may suggest that high level of sugar somewhat inhibits the algal growth, possibly due to the substrate inhibition, a common issue associated with batch cultures. The astaxanthin accumulation in *C. zofingiensis* is also related to the initial glucose concentration and enhanced astaxanthin content is achieved with high glucose concentration, e.g., the astaxanthin content at 30 g·L^−1^ glucose is 105% higher than that at 5 g·L^−1^ glucose [12], possibly due to that high glucose, when assimilated for glycolysis, provides more carbon precursors entering the astaxanthin biosynthetic pathway. It is worth noting, however, that further increases in sugar concentration fail to enhance astaxanthin level [7,12,104], indicating the presence of other limiting factors in addition to carbon precursors.

**Figure 8:**
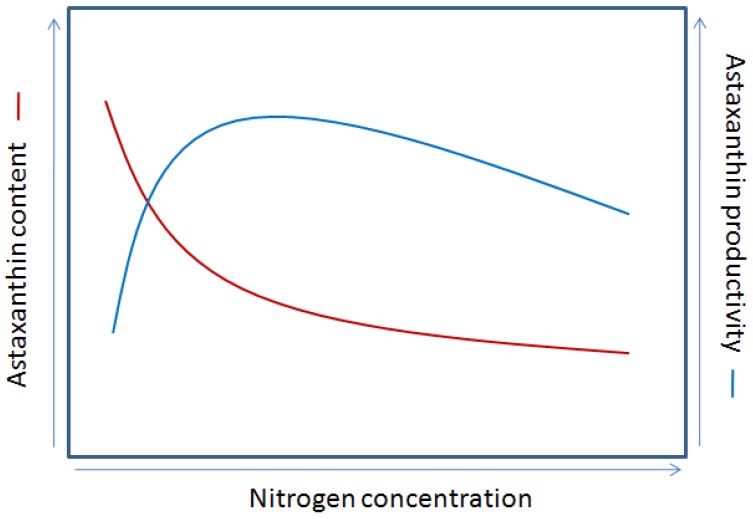
A schematic view of astaxanthin content and astaxanthin productivity as affected by nitrogen concentration *for C. zofingiensis*.

Nitrogen is a second important nutrient for algal growth and may account for up to 10% of the algal biomass. Commonly used nitrogen sources are in the form of nitrate, urea, and ammonia. Ammonia is economically more favorable than the other two nitrogen sources, but it tends to cause a drastic pH decrease, especially in unbuffered medium, and may result in severe growth inhibition [105,106]. In autotrophic cultures, nitrogen is an important factor influencing intracellular accumulation of secondary carotenoids, especially astaxanthin, and nitrogen limitation/starvation generally causes the enhanced synthesis of astaxanthin in *C. zofingiensis* [65,81]. However, nutrient limitation/deficiency can inhibit algal growth and may even lead to a complete cessation of growth. A prediction of astaxanthin content and astaxanthin productivity as affected by nitrogen concentration for *C. zofingiensis* is shown in Figure 8. By manipulating nitrogen concentration, the optimized astaxanthin productivity will be achieved in a batch culture. In heterotrophic cultures of *C. zofingiensis*, nitrogen availability plays also a critical role in astaxanthin accumulation. Considering organic carbons are used in heterotrophic cultures, carbon/nitrogen (C/N) ratio, controlling the switch between protein and lipid synthesis, is usually employed to show the combined effect of carbon and nitrogen on astaxanthin synthesis. Thus, it is the higher C/N ratios (corresponding to higher carbon concentrations when the initial nitrogen is fixed or lower nitrogen concentrations when the initial carbon is fixed) that trigger the accumulation of astaxanthin. For example, a C/N ratio of 180 gave rise to about one-fold increase in astaxanthin content as compared to a C/N ratio of 30 [7]. The increased astaxanthin may likely be due to the high C/N ratio providing excess carbon which enters into the carotenoid biosynthetic pathway.

Phosphorus is a third element required for normal growth of *C. zofingiensis*. The commonly used phosphorus is in a form of phosphate, either as H_2_PO_4_^−^ or HPO_4_^2−^. Phosphorus concentration, however, plays a much less prominent role on growth and astaxanthin content of *C. zofingiensis* than nitrogen, which is evident by the fact that phosphate at the range of 0.04–0.33 g·L^−1^ causes no significant change in specific growth rate, final cell density, or astaxanthin content [71].

Among the metal ions, iron is an important micronutrient for algal growth. It has been reported that iron deficiency caused a severe decrease in *C. zofingiensis* growth [71]. Iron also plays an important role in astaxanthin accumulation. The addition of ferrous ion was found to stimulate carotenogenesis in both *H. pluvialis* and *C. zofingiensis* for enhanced astaxanthin accumulation [107,108]. This may be explained, in part, by the fact that ferrous ion can serve as a generator of hydroxyl radical (·HO) through an Fe^2+^-catalyzed Fenton reaction in algal cells, and the accumulation of hydroxyl radical is believed to be capable of stimulating astaxanthin synthesis. Besides, ferrous ion is believed to be a cofactor for such enzymes as hydroxylases and ketolases involved in the biosynthesis of astaxanthin, and its presence may enhance the activity of these enzymes leading to increased astaxanthin levels [109]. Other metal ions, e.g., magnesium and manganese, have also been evaluated but are much less prominent in affecting astaxanthin synthesis as compared to iron [108].

#### 4.4.2. Environmental Factors

Photosynthesis requires light, which is indispensable for the photoautotrophic growth of algae. *C. zofingiensis* shows a typical light intensity-dependent growth. There is a saturation range of light intensity for optimal algal growth, below which the light becomes insufficient, leading to the photolimiation-associated slow growth; when exceeding the saturation value, photoinhibition occurs and causes growth attenuation or even cell death. It is worth noting that the saturation value may vary when different starting cell densities and/or optical light paths are employed. Astaxanthin biosynthesis can be greatly affected by light. It has been well studied in *H. pluvialis* that a high light intensity stimulates significantly enhanced astaxanthin accumulation [63,102,110]. *Similar to H. pluvialis*, *C. zofingiensis* is induced by high light to accumulate astaxanthin [11,65,66,83]. Considering that *C. zofingiensis* is capable of producing astaxanthin in the dark but at much lower rates [12,13], light is not necessary for triggering astaxanthin biosynthesis but for enhancing astaxanthin accumulation. The underlying mechanism is that high light irradiation produces excess photooxidation that could cause the generation of reactive oxygen species (ROS) such as singlet oxygen (^1^O_2_), superoxide anion (O_2_^−^), and hydroxyl radical (·OH); the antioxidative carotenoids and astaxanthin in particular are produced to protect the algal cells against oxidative damages of the ROS. Note that in most studies, high light is not deployed alone but accompanied by nitrogen limitation/starvation to induce carotenogenesis for astaxanthin accumulation [11,65,66]. These stress conditions, however, cause a severe reduction in algal growth and thus a reduction in the final astaxanthin yield of *C. zofingiensis*. To address the decreased growth and astaxanthin content, algal cells are usually cultured with replete nitrogen and a growth-favorable light intensity for optimal biomass production, followed by an induction stage with the stresses [6].

Salinity is another important environmental factor affecting astaxanthin accumulation in algae. Salt stress has been demonstrated to induce astaxanthin accumulation in both *H. pluvialis* and *C. zofingiensis* [11,111]. *C. zofingiensis* is a freshwater alga with a moderate tolerance of salt up to 0.1 M NaCl with just a slight impact on algal growth; further increase of salt concentration to 0.2 M and 0.4 M, however, reduces significantly the final cell density by 30% and 50%, respectively [11]. Nevertheless, addition of 0.2 M salt results in the highest astaxanthin yield, which is 60% higher than that of the salt-free cultures [11]. Similarly, Ip [71] reported little difference in cell density with the range of 0–0.08 M NaCl. However, the highest astaxanthin yield was achieved with 0.08 M NaCl; further increase of salt concentration caused severe decrease of the yield [71]. This difference may be explained by the different culture conditions used in these two studies.

Ip [71] also studied the effects of culture temperature and medium pH on growth and astaxanthin accumulation in *C. zofingiensis*. The alga grows well at the culture temperature of 20–30 °C and the optimal temperature for astaxanthin accumulation is between 25 and 30 °C. *C. zofingiensis* is capable of tolerating a relative wide range of pH (5.5–8.5) and the pH 5.5 gives rise to the highest astaxanthin content and astaxanthin yield. In addition, astaxanthin accumulation is enhanced by external supplementation of the culture medium with ROS such as hydrogen peroxide, or reactive nitrogen species/reactive nitrogen intermediates (RNS/RNI) such as peroxynitrite and nitryl chloride, or by the endogenous arousal of oxidative stresses via inhibition of intracellular antioxidative enzymes.

#### 4.4.3. Molecular Mechanism of Astaxanthin Biosynthesis

**Figure 9:**
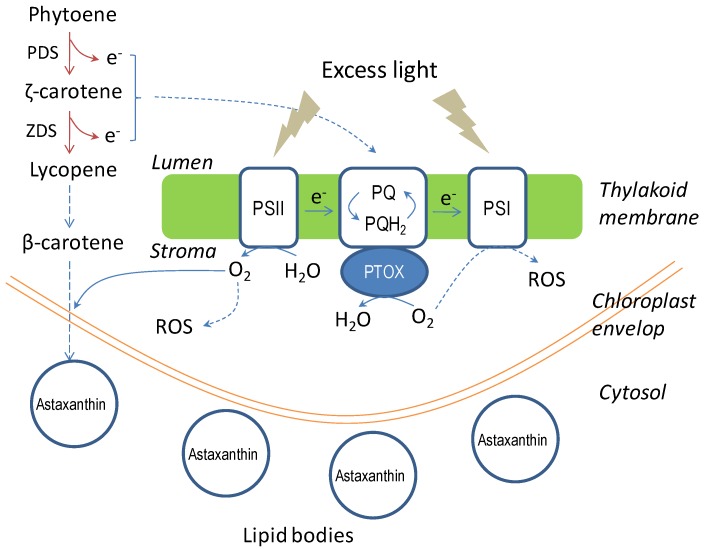
Illustration of the multiple roles of astaxanthin biosynthesis in protecting *C. zofingiensis* from photooxidative stress.

In *C. zofingiensis*, astaxanthin biosynthesis is believed to be regulated, at least in part, at the transcriptional level, as the mRNA level of carotenogenic genes is well coordinated with the intracellular content of astaxanthin. For instance, high light triggers the up-regulation of *CHYb* and *BKT*, and accordingly enhances the biosynthesis of zeaxanthin, canthaxanthin, and astaxanthin; while salinity stress up-regulates the expression of *BKT* only and thus promotes the biosynthesis of canthaxanthin and astaxanthin [83]. However, the 24–48 h delay between up-regulation of carotenogenic genes and the accumulation of astaxanthin indicate that they are not tightly coordinated and suggest that factors other than transcriptional control are involved in carotenoid biosynthesis. High light and salinity stresses stimulate the generation of ROS of different kinds, which may serve as the messenger molecule to trigger the expression of specific carotenogenic genes for enhanced astaxanthin accumulation. It is commonly perceived that the formation of astaxanthin is a survival strategy of the algae under photooxidative stress and other adverse conditions. Astaxanthin biosynthesis may offer multiple roles in protecting *C. zofingiensis* cells from those oxidative stresses (Figure 9), including (1) the accumulation of astaxanthin in cytoplasmic lipid bodies functioning as a “sunscreen” to reduce the amount of light impinging on chloroplast for attenuated photosynthetic photoinhibition and photodamage caused by excess photons; (2) lowering subcellular oxygen levels by forming oxygen-rich astaxanthin molecules; (3) converting photosynthetically evolved molecular oxygen into water via a concerted electron transport from carotenogenic desaturation steps through the photosynthetic plastoquinone pool (PQ) to plastid terminal oxidase (PTOX); (4) astaxanthin serving as an antioxidant to scavenge the generated ROS.

### 4.5. Mass Cultivation

*C. zofingiensis* is capable of growing autotrophically, mixotrophically, and heterotrophically. Generally, the mass cultivation of *C. zofingiensis* involves open systems, closed photobioreactors (PBRs), or fermenters. The *Chlorella* outdoor cultivation can be traced back to late 1940s with United States, Germany, and Japan launching cultivation processes almost concurrently. Circular pond is one of the most widely used open systems for *Chlorella* mass cultivation, and is reasonably scalable by increasing the pond diameter up to 50 m [112]. Raceway pond is another popular open culture system for *Chlorella* cultures. The ponds are usually maintained with cultures at a depth of less than 30 cm, aiming to facilitate the sunlight penetration to the bottom of the cultures [113]. Although both circular and raceway ponds are easy to construct and maintain in low cost, their drawback is that they can produce only very low cell densities (commonly less than 1 g cell dry weight per liter). Another open system, but rarely used, is the inclined thin layer cascade developed by Czech researchers [114]. This system characterizes itself by a thin layer of culture suspension, great flow turbulence, and high ratio of light-exposed surface area to volume, which together enable a high volumetric biomass yield (e.g., up to 40 g·L^−1^). This system may be more suitable than the circular and raceway ponds for growing *C. zofingiensis* for astaxanthin production, as it can provide a short light path and thus bestow excessive photons per cell to help induce astaxanthin accumulation. Nevertheless, the open systems possess intrinsic disadvantages such as rapid water loss, ease of contamination, and difficulty in controlling the cultures.

Closed photobioreactors (PBRs) can address the problems associated with the open systems. PBRs are made of transparent materials with a large surface area/volume ratio. Popular PBRs include tubular [113] and panel styles [115]. Feng *et al.* [14] tried to grow *C. zofingiensis* outdoors in panel PBRs, but achieved only a low volumetric biomass productivity, most likely due to the long light path of the PBRs. We deployed panel PBRs with various light paths and showed that the length of light path is critical to the biomass production of *C. zofingiensis* [116]. Within the light path range of 2–20 cm, the *C. zofingiensis* biomass productivity increases as the light path shortens, up to 1.2 g·L^−1^·day^−1^ (achieved with the light path of 2 cm); the short light path also benefits the accumulation of astaxanthin but this system requires high initial cell densities to minimize or eliminate photoinhibition. In this context, *C. zofingiensis* is first inoculated in panel PBRs with long light paths at low cell density to generate biomass. High-concentration cell cultures are then transferred to thin PBRs for continuing biomass production and astaxanthin accumulation.

**Figure 10:**
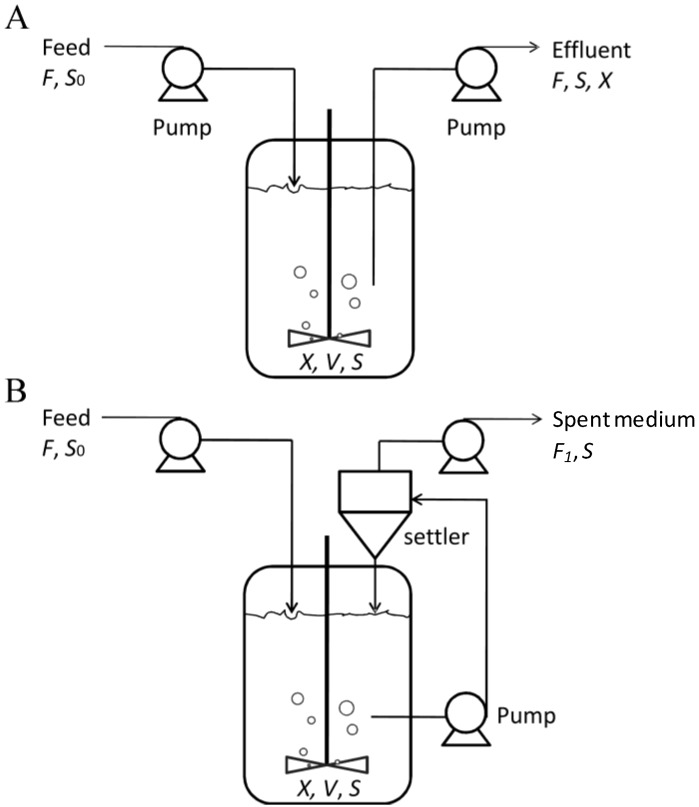
Schematic diagram of the continuous (**A**) and perfusion (**B**) fermenter culture systems. *X*, cell concentration; *V*, culture volume; *S*, carbon concentration in medium; *F*, flow rate of feed; *F*_1_, flow rate of perfusion; *S*_0_, carbon concentration in feed. The flow rates are controlled to maintain the culture volume constant.

*C. zofingiensis* can also grow well heterotrophically in fermenters [13,117]. Conventionally, fermenters are used for culturing non-photosynthetic organisms such as bacteria, yeasts, and animal cells. The well-developed fermenter technologies enable better control of *C. zofingiensis* growth for astaxanthin production. Generally, in a heterotrophic batch culture, a high initial concentration of substrates (e.g., glucose) is used to provide sufficient carbon for achieving a high cell density. However, the use of a high substrate concentration usually causes growth inhibition. For instance, a sugar concentration of over 30 g·L^−1^ inhibits the growth of *C. zofingiensis* [12,117]. The substrate-associated inhibition causes a decrease not only in the specific growth rate but also in the biomass yield coefficient based on sugars [13,117], thereby leading to increased cost inputs. To this end, a fed-batch cultivation strategy is deployed, which involves the stepwise addition of substrate to maintain algal cell growth with minimized inhibition. There have been several reports mentioning fed-batch cultivation of *C. zofingiensis* for astaxanthin production in which astaxanthin yield could reach up to 56 mg·L^−1^ [7,13,117]. Although the adoption of fed-batch culture strategy proves to be able to eliminate substrate-based inhibition, it is unable to overcome the inhibition caused by the toxic metabolites that are produced by the algal cultures and accumulate as the cells build up. To address this, other strategies may be applied, such as continuous cultivation with or without cell recycling. Continuous cultivation refers to the continuous addition of fresh medium with simultaneous removal of products to maintain a constant culture volume [118]. Figure 10A shows the schematic diagram of a continuous cultivation system. This system is capable of effectively eliminating metabolite-based inhibition but is accompanied by a drop in final cell density as well as sugar utilization efficiency. In contrast, continuous cultivation with cell recycling, known as perfusion culture, is a culture technique combining the advantages of both fed-batch and continuous culture system. Perfusion minimizes the inhibition associated with both high substrate concentration and buildup of toxic metabolites to maintain high cell density and productivity [119]. As illustrated by Figure 10B, in a perfusion culture system, the algal cells are retained by a retention device, whereas the spent medium (cell-free) is removed from the fermenter, with the simultaneous feeding of fresh medium to maintain sufficient nutrient supply. Though the intracellular astaxanthin content is significantly lower than that of photoautotrophic growth, heterotrophic growth offers much higher cell density and biomass productivity, thus giving rise to greater astaxanthin yield and astaxanthin productivity as well [7,13,66].

**Table 2:** Production of biomass and astaxanthin by *C. zofingiensis* and other organisms.

Strain	Culture Conditions ^a^	Cell Density (g·L^−1^)	Biomass Productivity (g·L^−1^·day^−1^)	Astaxanthin Content (mg·g^−1^ Dry Weight)	Astaxanthin Yield (mg·L^−1^)	Astaxanthin Productivity (mg·L^−1^·day^−1^)	References
*C. zofingiensis*							
CCAP211/14	P, batch	6.7	0.74	3.7	24.8	2.8	[11]
SAG211/14	P, batch	-	-	6.5	-	0.8	[66]
ATCC30412	M, batch	9.5	-	1.3	12.5	-	[104]
ATCC30412	H, batch	10.2	-	1.0	10.3	-	[12]
ATCC30412	H, fed-batch	53	3.5	0.69	32.4	2.2	[13]
ATCC30412	H, batch	12.9	1.6	1.18	13.6	1.7	[117]
ATCC30412	H, batch	10.3	2.1	1.31	13.5	2.3	[7]
	H, fed-batch	45.6	4.7	1.19	56.1	5.6	
*H. pluvialis*							
CCAP34/7	P, batch	1.6	0.02	27	43.2	0.44	[111]
UTEX16	M, batchM, fed-batch	2.652.74	0.130.14	20.123.5	53.464.4	2.63.2	[6]
CCAP34/7	P, batch	-	-	22.7	-	2.7	[66]
CCAP34/8	P, batch	7.0	0.41	11.0	77	4.4	[120]
CCAP34/8	P, continuous	-	0.6	8	-	5.6	[121]
NIES-144	P, fed-batch	6.7	0.2	36	390	7.2	[122]
CCAP34/8	P, continuous	1.5	0.7	10	15	7	[123]
*X. dendrorhous*							
ATCC24202	H, fed-batch	30	5.1	0.72	21.6	3.7	[124]
NRRL Y17268	H, Batch	23.2	3.4	0.45	10.4	1.5	[125]
ATCC24202	H, fed-batch	18.8	4.7	0.3	5.7	1.4	[126]
2A2N ^b^	H, batch	36	7.2	1.1	39.6	7.9	[127]
ZJUT46 ^b^	H, batch	15.7	2.85	1.74	27.1	5.0	[128]
	H, fed-batch	17.7	3.2	2.0	34.4	6.4	[129]
E5042 ^b^	H, batch	30.7	5.4	2.5	76.8	13.5	[130]
VKPM Y2476 ^b^	M, fed-batch	88	9.7	4.7	420	45.6	[131]

^a^ P, photoautrophic, M, mixotrophic, H, heterotrophic; ^b^ mutants.

### 4.6. Comparison of Astaxanthin Production from C. zofingiensis and Other Organisms

The green microalgae *H. pluvialis* and *C. zofingiensis*, and the yeast *X. dendrorhous* are the most studied microorganisms for astaxanthin production. In general, microbe-based astaxanthin production pipeline involves a set of processes: astaxanthin-rich biomass production, harvest, drying, cell disruption and astaxanthin extraction, and concentration and purification if necessary. Astaxanthin content is a key factor to access the suitability of astaxanthin production from a selected organism, and higher astaxanthin content is considered more cost favorable for downstream process. In this context, among the three organisms, *H. pluvialis* has been the first choice as a source for natural astaxanthin, followed by *C. zofingiensis* and *X. dendrorhous*, as *H. pluvialis* has the highest intracellular astaxanthin content that may reach up to 3.6% of dry weight (Table 2). *X. dendrorhous* possesses the lowest level of astaxanthin, which is commonly below 0.1% of cell dry weight; it is worth mentioning, however, that some *X. dendrorhous* mutants can accumulate significantly more astaxanthin than the wild type ones (Table 2). Biomass density is also an important factor and high density helps to lower the harvest cost, which may contribute up to 30% of the overall cost depending on the organism used and the biomass concentration. *H. pluvialis* grows slowly and achieves only a low cell density (e.g., up to 7 g·L^−1^); in contrast, *C. zofingiensis* grows fast and can build up an ultrahigh cell density (with a potential of up to 100 g·L^−1^), thereby greatly lowering the harvest cost as compared to *H. pluvialis*. With the high cell density, the volumetric astaxanthin yield and productivity of *C. zofingiensis* are comparable to that of *H. pluvialis* (Table 2). What is more, unlike *H. pluvialis* or *X. dendrorhous*, *C. zofingiensis* is capable of growing robustly under both heterotrophic (high cell density) and photoautotrophic (enhanced astaxanthin content) conditions, which may warrant further exploration of this alga for astaxanthin production.

## 5. Possible Improvements in *C. zofingiensis* Astaxanthin Production Economics

*C. zofingiensis* has shown great potential as an alternative source of astaxanthin, and the astaxanthin production economics of *C. zofingiensis* can be further improved by strain improvement via genetic engineering, development of next generation culture systems, and the establishment of biorefinery production strategies.

The relatively low intracellular astaxanthin content is a key issue contributing to the relatively high production cost of *C. zofingiensis* astaxanthin. Mutagenesis has been demonstrated as an effective approach to enhancing the biosynthesis of carotenoids for elevated astaxanthin accumulation in *H. pluvialis* [68,132]. Recently, enhanced astaxanthin content has also been achieved in *C. zofingiensis* by mutagenesis [133]. Comprehensive screening with further mutagenesis may produce mutants with even higher astaxanthin content. Compared to the time-consuming mutagenesis methods, direct genetic engineering of the astaxanthin pathway appears to be a more feasible and efficient approach to increasing the intracellular astaxanthin accumulation in *C. zofingiensis*. As indicated by Figure 7, in *C. zofingiensis*, astaxanthin accounts for *ca.* 50% of total keto-carotenoids with the other 50% mostly adonixanthin and canthaxanthin. This limits its use for humans as such mixtures are not approved in the USA, Europe or Australia either for health foods, functional foods or other applications such as food coloring. A purification process, which is challenging and adds cost to astaxanthin production, is therefore needed, making *C. zofingiensis* currently less favorable. Considering that the *C. zofingiensis* CHYb cannot accept canthaxanthin as the substrate to produce astaxanthin and the BKT has a relatively low activity for catalyzing the formation of astaxanthin from adonixanthin, manipulating the specific astaxanthin biosynthetic steps should largely promote the percentage of astaxanthin (purity) and enhance the astaxanthin production on a per cell basis (content). This engineering of astaxanthin production could be achieved by introducing a CHYb that can convert canthaxanthin to astaxanthin and a BKT that can catalyze the efficient conversion of adonixanthin to astaxanthin using an efficient and stable transformation system for *C. zofingiensis* recently developed [69]. 

Photobioreactors are believed to be a promising system for photoautotrophic mass cultivation of *C. zofigniensis*. However, there are many issues associated with the current PBR system, such as relatively poor light availability and poor mixing when scaling up, buildup of dissolved oxygen, large dark volumes, and the intensive capital required for construction and maintenance. The development of next generation PBR systems addressing the above mentioned drawbacks will have great potential to improve the production economics. Furthermore, PBRs can be coupled with fermenters for growing *C. zofingiensis* and astaxanthin production. A sophisticated fermenter system bestows great advantages for biomass production of *C. zofingiensis*, but the intracellular astaxanthin content in this system needs to be improved. For instance, the maximal astaxanthin content achieved in heterotrophic cultures is below 2 mg·g^−1^, while in autotrophic cultures provided with high light, the astaxanthin can reach up to 6 mg·g^−1^ [7,11,13,66], indicating that light is a key factor for astaxanthin induction and accumulation. In this context, we propose a heterotrophic-phototrophic two-stage culture strategy to grow *C. zofingiensis* to improve production of astaxanthin: *C. zofingiensis* is first cultured heterotrophically in fermenters for rapid accumulation of biomass which is then transferred to PBRs with high light conditions for the induction of astaxanthin. The proposed culture strategy combines the advantages of both heterotrophic and phototrophic modes and eliminates the possible contamination associated with mixotrophic growth, and can enhance astaxanthin production by three-fold [116]. This may open a door to substituting the currently used heterotrophic culture method for scale-up toward possible commercial production of astaxanthin by *C. zofingiensis*.

*C. zofingiensis* also produces substantial amounts of lipids, carbohydrates, and proteins. From a biorefinery perspective, the lipids are suitable for biofuels production and the residual biomass after lipid extraction can be potentially used as nutraceuticals and animal feeds. In addition, the carbohydrates may be utilized to produce methane by anaerobic digestion. The integrated production of astaxanthin, oil, and other products, coupled with the possible recycling of water and nutrients, represents a potential and promising strategy toward further improving *C. zofingiensis* astaxanthin production economics.

## 6. Conclusions

Microalgae represent a promising source of natural astaxanthin that can potentially replace the chemically synthesized astaxanthin. *C. zofingiensis*, a possible alternative producer for astaxanthin, has been intensively investigated in recent years. To date, substantial achievements have allowed better understanding of astaxanthin biosynthesis in *C. zofingiensis* and should soon allow utilization of this alga for astaxanthin production. Future exploration of using *C. zofingiensis* for astaxanthin production may lie in genetic engineering of this alga for enhanced biosynthesis of astaxanthin (to increase astaxanthin content and purity), development of next generation culture systems for sustainable and cost-effective production of astaxanthin-rich biomass, and exploration of state-of-art biorefinery-based integrated production strategies. Advances occurring in these areas will greatly expand the production capacity and lower the production cost, allowing *C. zofingiensis* to be a competitive source of natural astaxanthin. To this end, “omics” analyses (genomics, transcriptomics, and proteomics) of *C. zofingiensis* are currently underway, which will benefit the understanding of astaxanthin biosynthesis and development of a new molecular toolbox for more efficient manipulation of the production strain toward economically feasible production of astaxanthin.
